# Combined functional and structural imaging of brain white matter reveals stage-dependent impairment in multiple system atrophy of cerebellar type

**DOI:** 10.1038/s41531-022-00371-2

**Published:** 2022-08-17

**Authors:** Hua Lin, Li Lin, Lyuan Xu, Siran Li, Penghui Song, Muwei Li

**Affiliations:** 1 Department of Neurology, Xuanwu Hospital, Capital Medical University, Beijing, 100053 China; 2 Department of Radiology, the Third Affiliated Hospital of Sun Yat-sen University, 600 Tianhe Road, Guangzhou, Guangdong Province 510630 China; 3 Vanderbilt University Institute of Imaging Science, Vanderbilt University, Nashville, TN USA; 4 Department of Radiology and Radiological Sciences, Vanderbilt University Medical Center, Nashville, TN USA

**Keywords:** Movement disorders, Neurodegenerative diseases

## Abstract

Advances in fMRI of brain white matter (WM) have established the feasibility of understanding how functional signals of WM evolve with brain diseases. By combining functional signals with structural features of WM, the current study characterizes functional and structural impairments of WM in cerebelar type multiple system atrophy, with the goal to derive new mechanistic insights into the pathological progression of this disease. Our analysis of 30 well-diagnosed patients revealed pronounced decreases in functional connectivity in WM bundles of the cerebellum and brainstem, and concomitant local structural alterations that depended on the disease stage. The novel findings implicate a critical time point in the pathological evolution of the disease, which could guide optimal therapeutic interventions. Furthermore, fMRI signals of impaired WM bundles exhibited superior sensitivity in differentiating initial disease development, which demonstrates great potential of using these signals to inform disease management.

## Introduction

Multiple system atrophy (MSA) is an adult-onset progressive neurodegenerative disease characterized by parkinsonism, cerebellar ataxia and autonomic dysfunction with additional neurological symptoms^1^. Depending on the region of neurodegeneration, MSA can be broadly divided into predominant cerebellar type (MSA-C) or parkinsonian-type (MSA-P)^2^. The neuropathological hallmark of MSA is progressive accumulations of α-synuclein-containing glial cytoplasmic inclusions in oligodendrocytes^3,4^, which are remarkably abundant in white matter (WM). While diagnoses of MSA and assessments of the severity of its consequent disabilities conventionally resort to subjects’ performance evaluations^5^, modern neuroimaging techniques particularly diffusion tensor imaging (DTI) have been widely utilized during the past decades to non-invasively probe pathological alterations of the neuronal pathways involved in MSA^6^. Indeed, DTI possesses the sensitivity of detecting microstructural WM abnormalities in regions that would otherwise appear normal by routine magnetic resonance imaging (MRI), thus offering the potential of deriving mechanistic insights into the pathogenesis and evolution of MSA. However, there is a lack of information on the functional changes in MSA-C, especially in the early stages.

It has been well established from neurophysiology that brain structure is intimately related to function. A natural question on MSA then arises regarding how the impairments to WM microstructure alter its function, which will impart on relevant gray matter (GM) function and eventually lead to clinical endpoints^7^. While WM function serves as a key link between WM structure and GM function, only recently the potential of detecting WM function non-invasively with functional MRI (fMRI) has been demonstrated^8^(also see reviews in the Gore et al.^9^ and Grajauskas et al.^10^). Historically, measuring WM function in vivo has been very challenging, presumably attributable to the sparse vasculature that irrigates WM^11^ and accordingly low oxygen metabolism therein^10^. The difficulty has been largely ameliorated by using appropriate methods that allow sensitive and robust detections of functional activities in WM^12–14^. For instance, by clustering fMRI signals, Peer et al. identified several WM functional networks that correlate specifically with GM networks^15^. Similarly, based on independent component analysis, Huang et al. were able to parcellate WM into distinct structures that coincide with corresponding tracts obtained from DTI^16^. In addition, it is observed that functional loading to the brain enhances functional activities in WM^17,18^ whereas anesthesia tends to reduce them^19^, and local glucose metabolisms increase with functional activities in WM^20^. Moreover, by using stereo- electroencephalography, it has been confirmed very recently that motor tasks can activate WM which also participates in movement encoding, thus providing a direct evidence of WM activation by neural events^21^. Taken together, these findings support the notion that fMRI signals in WM encode neural activities, which may be reliably detected to yield more complete understanding of brain structure-function relations and to study how they evolve with development, aging or pathological processes. In fact, recently a wide variety of clinical applications have been developed to look into WM functional activity changes as a result of neurological or psychiatric disorders^22–32^, which have brought in growing evidences at the clinical end that further confirm the detectability of WM function and its practical utility.

The motivation of this work is to make full use of the current advances in WM fMRI to explore the possibility of characterizing functional alterations in the WM of patients diagnosed as the subtype MSA-C, which is the first work of this kind in this research area. Our primary goal is to identify WM structures that exhibit reduced functional activities due to the disease process and to examine the relationship of functional measures to those of tissue microstructure. We will classify the MSA-C patients into early- and nonearly-stages on the basis of disease duration, and assess the potential of utilizing the functional signals in WM as an additional biomarker to enhance the performance of conventional diagnosis, particularly for early-stage patients.

In the following, we debut our investigations outlined above. First, we parcellate the brain volume into distinct GM and WM regions based on standard brain atlases, and compute pairwise temporal correlations in fMRI signals between the GM and WM parcels, using the approach proposed by Ding et al.^33^. The fMRI signals are obtained from a cohort of early- and nonearly-stage MSA-C patients and matched normal controls (NCs) with resting-state acquisitions, and thus the computed functional correlations reflect coordinated intrinsic neural activities across different brain regions. We then present our detailed analysis of functional correlation profiles in the WM of MSA-C patients and compare them with NCs. Relations of WM functions to microstructure will be examined by comparing functional measures from fMRI with structural metrics derived from DTI. Finally, we report performances of diagnosing MSA-C patients using discriminant analysis of fMRI signals.

## Results

### Demographics and clinical variables of the patients

The study included 30 patients diagnosed with MSA-C and 19 NCs. The MSA-C patients and NCs had no significant differences in age and sex (both *P* > 0.05). Demographic and clinical characteristics of the MSA-C patients are summarized in Table 1. Seventeen patients (57%) of the 30 were males and 13 (43%) were females. Mean age at symptom onset was 55.0 (SD 6.4) years; mean duration of symptoms was 2.7 (SD 1.7) years. According to consensus criteria^1^, 19 patients (63%) fulfilled criteria for probable MSA (autonomic failure) and 11 patients (37%) for possible MSA (autonomic dysfunction). All patients with MSA-C had cerebellar symptoms, and 19 (63%) had orthostatic hypotension. Twelve patients presented orthostatic discomfort such as dizziness resulting from hypoperfusion, and syncope likely occurred in 2 patients. Urinary symptoms were more common including incomplete bladder emptying (25 [83%]) and urinary incontinence (5 [17%]). Erectile dysfunction affected virtually most male patients, which is often the earliest symptom of MSA.

**Table 1:** Demographic characteristics and clinical measures of MSA-C patients.

Category	Characteristic	Value
Demography		
	Number	30
	Age (years)	57.6 ± 7.3
	Early-stage	54.4 ± 7.6
	Nonearly-stage	60.3 ± 5.0
	Sex	17 males, 13 females
	Disease duration (years)	2.7 ± 1.7
	Early-stage	1.34 ± 0.57
	Nonearly-stage	4.07 ± 1.33
	Age of onset (years)	55.0 ± 6.4
Clinical measures		
	UMSARS-I	13.7 ± 7.5
	UMSARS-II	10.9 ± 6.0
	SARA	13.1 ± 7.7

To identify early functional-structural abnormalities in MSA, patients were divided into two subgroups: early-stage MSA-C (disease duration ≤2 years) and nonearly-stage MSA-C (disease duration >2 years). Among all the 30 MSA-C patients, 15 (50%) were in early-stage (duration range = 0.5–2 years) with the other 15 (50%) in nonearly-stage (duration range = 3–8 years). The average age of the early-stage and nonearly-stage subgroups was 54 (range = 41–63) and 60 (range = 46–69) years respectively, with the early-stage patients being younger than those of the nonearly-stage group (*P* < 0.05). Among the 15 early-stage patients, 8 (53%) were diagnosed for probable MSA-C and 7 (47%) for possible MSA-C. Among the 15 nonearly-stage patients, 11 (73%) were diagnosed for probable MSA-C and 4 (27%) for possible MSA-C.

### Temporal correlation analysis of fMRI signals in WM

**Fig. 1:**
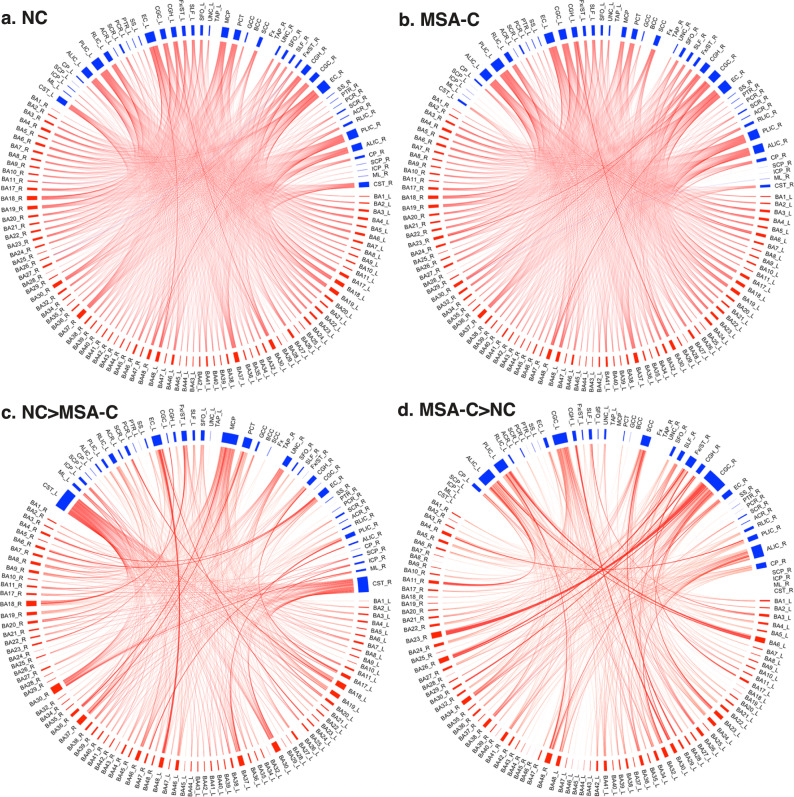
Brain connectome of pairwise temporal correlations between parcellated WM bundles and GM regions. **a** Connectome of the NC group. **b** Connectome of the MSA-C group. **c** Connectome of differences between (A) and (B) where correlations in the NCs greater than those in the MSA-Cs. **d** Connectome of differences between **a** and **b** where correlations in the NCs smaller than those in the MSA-Cs. The WM bundles (denoted as blue boxes) are defined using the JHU WM atlas, and the GM regions (red boxes) are parcellated using Brodmann’s definitions. The thickness of the curve represents the strength of Pearson correlations in fMRI signals between a WM-GM pair, which is computed by averaging the correlation coefficients over the entire group (negative correlations are represented by blue curves, which are hardly visible due to their small values). It can be appreciated that, overall, WM has more and greater correlation differences in **c** than **d**, suggesting that the disease tended to impair functional signals in most of the WM bundles. See Supplementary Material A for the list of abbreviations for the WM bundles. Note that _L and _R means the left and right hemisphere respectively.

Pairwise temporal correlations between WM bundles and GM regions for the MSA-C and NC groups are shown in Fig. 1a, b, in which red boxes denote the 48 WM bundles and blue boxes denote the 82 GM regions used in the study. Each curve represents the correlation coefficient (CC) of fMRI signals between a WM-GM pair, averaged over the entire group, with thickness proportional to the average correlation strength. Overall, WM bundles in the disease group tended to have smaller correlations with GM regions than the control group, as can be appreciated from the bottom row of Fig. 1, which contains much fewer connections in (d) than in (c).

**Fig. 2:**
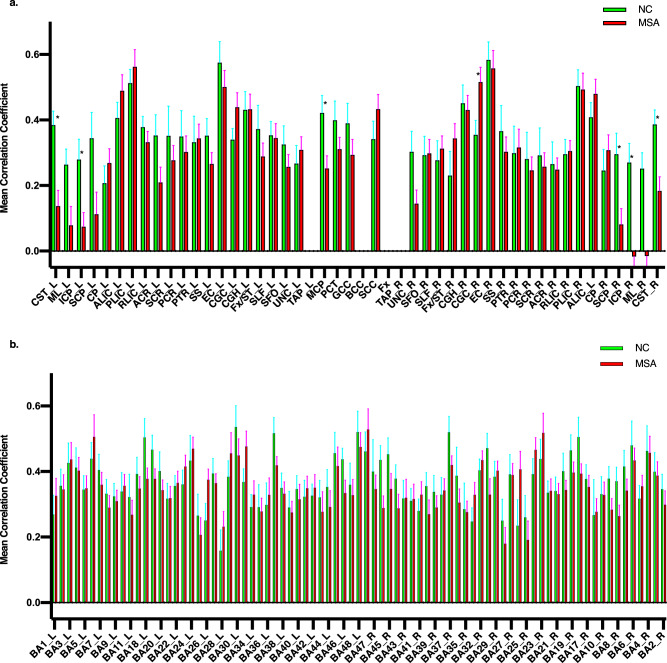
Comparisons of temporal correlations of white matter and gray matter fMRI signals between normal controls and MSA-C patients. Comparisons of averaged temporal correlations of WM bundles (**a**) and the GM region (**b**) between the control (green) and disease (red) groups. Mean and standard error are shown, with significant differences (*P* < 0.05, corrected) marked with asterisks (*).

Figure 2a shows the mean CC (mCC) for each of the 48 WM bundles studied, defined as the CC of a WM bundle averaged over all its GM correlations, for the MSA-C (red) and NC (green) group. As can be seen, the majority of the WM bundles had a smaller mCC in the disease group than in the control group. Bundle-wise *t* tests found that, compared to the NCs, the patients had significantly lower mCC in the left and right corticospinal tract (CST), bilateral inferior cerebellar peduncle (ICP), middle cerebellar peduncle (MCP), right superior cerebellar peduncle (SCP), but significantly greater mCC in the right cingulum (*P* < 0.05). These significances still survived after false-positive correction with *P* < (1/48). Note that the mean CC of each of the 82 GM regions (averaged over the 48 WM bundles) was also computed (Fig. 2b), but none of them had a significant difference between the two groups.

Moreover, to provide some mechanistic insight into the observed abnormalities in functional connectivity, mean power spectrum density (PSD) of fMRI signals in the WM bundles identified above also examined, with results plotted in Supplementary Fig. 1a–d.

### Structural analysis of WM bundles in MSA-C patients using DTI

**Fig. 3:**
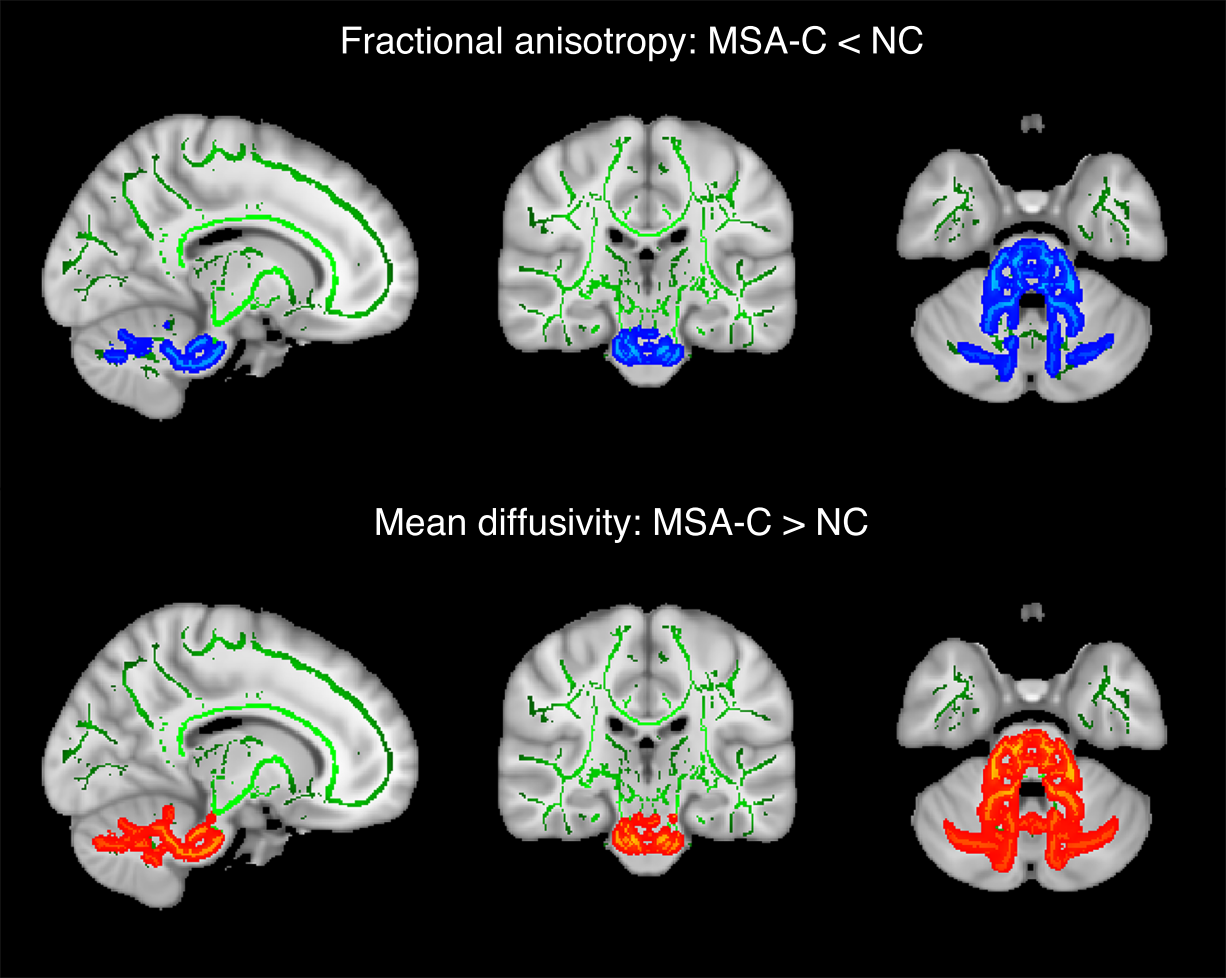
Comparisons of fractional anisotropy and mean diffusivity between normal controls and MSA-C patients. Green curves are the skeletons of white matter tracts obtained from analysis of tract-based spatial statistics. Blue curves in the top row show portions of the skeletons where mean fractional anisotropy in the disease group was significantly smaller than the control group (*P* < 0.01, TFCE and FWE corrected), and red curves in the bottom row show portions of the skeletons where mean diffusivity in the disease group was significantly greater than the control group (*P* < 0.01, TFCE and FWE corrected). Note that a representative image slice is visualized, respectively, for sagittal, coronal and axial view, and the image right is the subject left in the second and third column.

To further identify WM structures that exhibit reduced functional activities due to the disease process, microstructural measures derived using TBSS (Tract-Based Spatial Statistics) analysis on DTI data are visualized in Fig. 3, which displays a sagittal (left), coronal (middle) and axial (right) view of a representative slice each. The top row shows the voxel locations (blue colored) where the MSA-C patients had significantly smaller fractional anisotropy (FA) than the NCs (two-tailed *t* tests, *P* < 0.01, TFCE and FWE corrected), and the bottom row shows the voxel locations (red colored) where the patients had significantly greater mean diffusivity (MD) (two-tailed *t* tests, *P* < 0.01, TFCE and FWE corrected). No significant differences along the opposite direction were observed for FA and MD. As expected, the differences in FA and MD were found to prevalently co-occur in the same set of WM bundles, including the MCP, pontine crossing (PC) tract, right SCP, left CST, left, and right ICP.

**Fig. 4:**
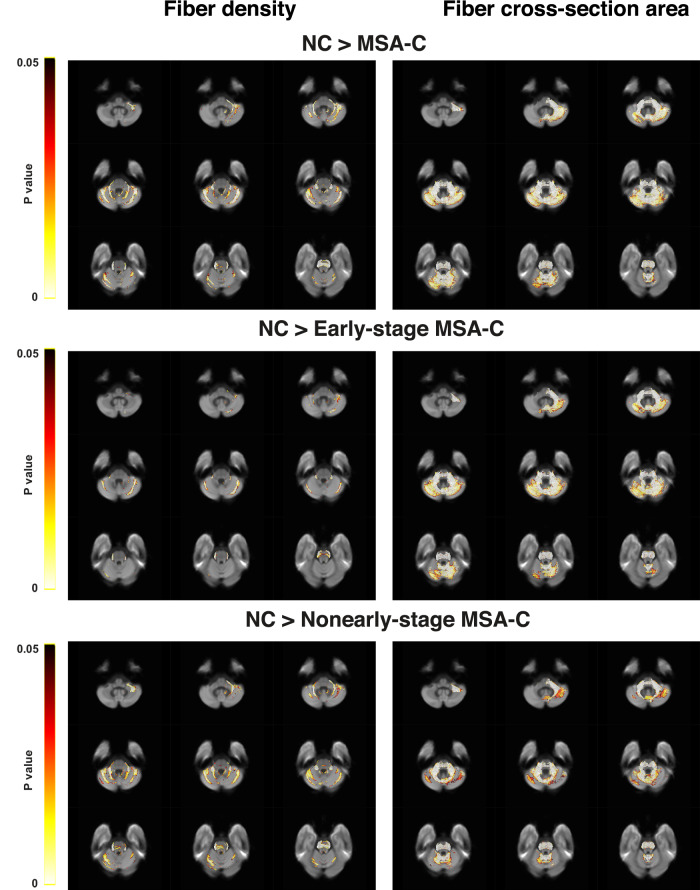
Comparisons of fiber density and bundle cross-sectional area between the MSA-C patients and normal controls. Indices of fiber density and bundle cross-sectional area were derived using fixel-based analysis described in Supplementary Material A. In each voxel location, *p* value is the average of the *p* values from all the fixels it contains. *p* values of significant differences between the two groups are color coded and superimposed onto representative 2D anatomical slices. Panels from top to bottom show respectively the voxel locations with significant decrease (*P* < 0.05, FWE corrected) in fiber density (left column) and bundle cross-sectional area (right column) in all the MSA patients, early- and nonearly-stage patients relative to the controls. Radiologic view conventions are used.

Voxel locations with significantly reduced fiber density and bundle cross-sectional area are shown in the left and right column of Fig. 4 respectively, in which the color encodes the *p* value of the difference between the patients and controls (FWE corrected). Visual inspections reveal that the patients had significantly decreased within-voxel fiber density and macroscopic bundle cross-sectional area mostly in the brainstem and cerebellum (top panel), anatomically consistent with the observations from our fMRI analysis. Note that the decrease in bundle cross-sectional area spanned much wider WM regions than the fiber density, indicating that fiber dimension reductions were a more widespread pathology than axonal losses in the MSA-C patients.

Detailed comparisons between the two patient subgroups found that the early-stage patients had more prominent decrease in fiber-bundle cross-sectional area than the nonearly-stage counterparts, whereas the latter had several times more decrease in fiber density (see Table 2 for quantitative comparisons). The stage-dependent difference in the structural measures of WM bundles reflected that, as the disease evolved, the pathological processes changed from dimension reductions of fiber bundles mainly at the beginning to heavy axonal losses finally, the latter of which represents drastically more severe pathological conditions.

**Table 2:** Quantitative analysis in fiber-bundle cross-sectional area and fiber density of MSA-C.

Structural measure	All MSA-C	Early-stage	Nonearly-stage
Fiber-bundle cross-sectional area	33,663 (91%)	28,158 (97%)	19,511 (80%)
Fiber density	8283 (22%)	2581 (9%)	8788 (36%)
Total	37026	29001	24279

The numerals indicate the number of voxels with significant difference and the percentage is with respect to the total number of fixels. See Supplementary Fig. 2. for superimposed distributions of fiber-bundle cross-sectional area and fiber density.

### Classifications of MSA-C patients

**Fig. 5:**
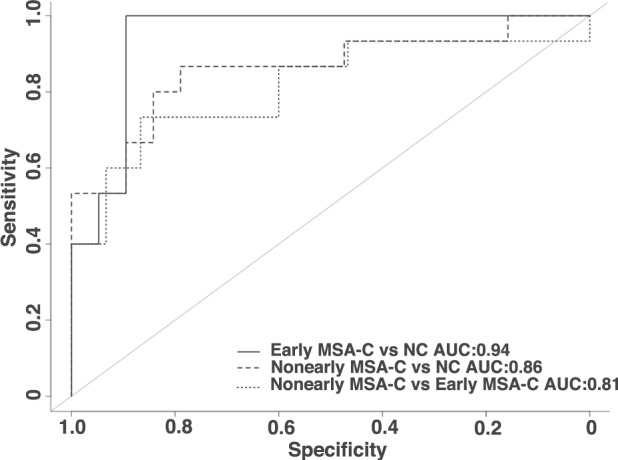
Receiver operating curves of the classification models for discriminating MSA-C and NC. Different models are plotted with different line styles. Discrimination between the early-stage patients and NCs achieved the best performance among the three models.

Finally, to assess the potential of utilizing the functional signals in WM as a new neuroimaging biomarker, the mCCs of the seven abnormal WM bundles identified above were used as features for our support vector machines (SVM) classifier to discriminate among the early- and nonearly-stage MSA-C patients as well as the NCs, with performances summarized in Table 3 and the corresponding receiver operating curves drawn in Fig. 5. Among the three classification models, the discrimination between the early-stage patients and NCs achieved the highest performance of 0.94, 100%, and 89% for area under the curve (AUC), sensitivity and specificity, respectively. The discrimination between the nonearly-stage patients and NCs achieved moderately high performance of 0.86, 87%, and 79% for AUC, sensitivity and specificity respectively, followed by 0.81, 87%, and 73% for the three performance measures in discriminating between early- and nonearly-stage patients. The good discrimination power demonstrates that functional signals in the abnormal WM bundles could effectively differentiate diseased and healthy subjects as well as early- and nonearly-stage MSA-C patients. Notably, the 100% sensitivity in discriminating the early-stage patients from NCs has particular implications to early diagnosis and management of this disease.

**Table 3:** Performances of discriminative analysis with support vector machine.

Classification model	Area under curve	Sensitivity (%)	Specificity (%)
Early MSA-C vs NC	0.94	100	89
Nonearly MSA-C vs NC	0.86	87	79
Early vs nonearly MSA-C	0.81	87	73

Additionally, to explore whether WM functional signals could predict subject’s behavior, we performed stepwise linear regression analyses between mCC of the seven abnormal WM bundles and each of the clinical measures collected (UMSARS-I, UMSARS-II and standardized scale for the assessment and rating of ataxia; SARA) individually for both the disease subgroups. There was a significant relationship for the early-stage patients (see Supplementary Material E).

## Discussion

Recent advances in fMRI of brain WM have permeated many clinical studies aimed at understanding structure-function relations of WM, the manner in which they evolve with brain disease, and how functional impairments to WM are associated with clinical manifestations^23,26,29^. While existing clinical studies to date are focused on neurological or psychiatric disorders mainly affecting cerebral WM, the current work extends them into the domain of cerebellum and brainstem. We performed detailed region-wise characterizations of WM functional alterations in patients with MSA-C, interpreted WM functions from the perspective of tissue structure and explored the potential of using WM fMRI signals as indicators of brain dysfunction. Pronounced signatures of WM functional abnormalities were observed, which primarily affected the cerebellum and brainstem and CSTs that connect them to the cortex. Our additional power spectrum analysis revealed that patients with MSA-C had markedly altered spontaneous oscillations in the WM regions impacted by the disease. Furthermore, tensor- and fixel-based analyses using diffusion imaging indicated micro- and macrostructural changes of WM that were in keeping well with fMRI findings. More notably, the changes in WM micro- and macrostructure were found to be dependent on disease stage, which implicates a critical time point that defines pathological progressions of the disease. Of particular clinical relevance, signatures of WM functional abnormalities exhibited high sensitivity and specificity in identifying early-stage MSA-C patients, and the correlation observed between WM functional signals and clinical scores suggests that the signals encode information on human behavior, particularly during the early phase of the disease, which could be exploited for clinical purposes. Overall, this study provides an in-depth, comprehensive and previously unavailable view of WM structure and function and the relation between them in MSA-C patients, and offers the potential of developing neuroimaging biomarkers to assist in early diagnosis of this disease.

As evident from our analysis, the WM regions impacted by the MSA-C disease are primarily in the brain motor circuitry. Previously, it has been demonstrated that motor tasks can modulate functional signals in motor system WM of healthy human subjects^34,35^, including modulations of the signal power spectra^36^. The current study further investigated alterations of WM functional signals under pathological conditions, which represents a first effort in this area. We found that, compared to healthy controls, MSA-C patients had a significantly lower functional connectivity (i.e., functional correlation) in several WM bundles, including the inferior, middle and superior cerebellar peduncle, and CSTs, which agree well with previous DTI studies in MSA-C^37^. Of a particular note, the right cingulum exhibited an increased functional connectivity in the patients. As this bundle is not a part of the motor circuitry, the observed increase in its functional connectivity may serve as a mechanism to compensate for disease induced functional impairments to WM bundles in the motor system. The abnormalities in functional connectivity of the WM bundles were accompanied by alterations in the frequency domain of the functional signals. We found that the WM bundles in these patients exhibited qualitatively differences in PSD profiles, and these differences depended on the duration of the disease. Taken together, the decreased functional connectivity and altered spontaneous signal oscillations observed in this study are indicative of impaired communications in the WM bundles involved. Metabolically, it has been found^38^ that MSA-C patients exhibited a reduced glucose uptake rate in the cerebellum, pons and medulla, and a hypometabolism in the cerebellum and brainstem, along with prefrontal and cingulate cortices, for those characterized by cerebellar and laryngeal-pharyngeal involvement symptoms^39^. Since neural activities necessarily incur local metabolic demands, these brain metabolic reductions converge with our findings of reduced WM functional connectivity in the MSA-C patients.

The identification of functionally abnormal WM bundles provides a basis for explorations of structural and pathological mechanisms for disease evolution. Recent volumetric studies of MSA revealed that brain atrophy mostly involved the cerebellum and brainstem^37^, which are presumably consequential to WM loss therein. Meanwhile, our diffusion imaging studies found concomitant FA decrease and MD increase at the inferior, middle and superior cerebellar peduncle, pontine crossing tract and CST. The convergent findings from the previous volumetric and our diffusion along with functional imaging studies suggest that the impaired WM functionalities in the MSA-C patients were related to compromised structures. Biopathologically, the hallmark of MSA is accumulation of a-synuclein–containing inclusions in oligodendrocytes^4,40^, which are mainly concentrated in WM including the projection pathways, corpus callosum, anterior commissure, pyramidal tract, pons, MCP, cerebellar WM, and the reticular formation.^4^ Aggregations of α-synuclein in oligodendrocytes^41^ in principle signify structural damages to the WM regions^42^, which may underlie the functional abnormalities we observed. It should be noted that WM morphometric changes may involve fiber density or fiber-bundle dimension changes or both^43^. Our analysis showed that MAS-C patients had significant reductions of regional WM volumes manifested as decreases in both within-voxel fiber density and macroscopic fiber-bundle cross-section size, which, much to our expectation, were maximal in the WM of brainstem and cerebella. These findings are consistent with those using fixel-based analysis of MSA^44^. Furthermore, it was found that the early-stage MSA-C patients (disease duration ≤ 2 years) exhibited greater decreases in fiber-bundle size, whereas reduced fiber density was more prominent in the nonearly-stage MSA-C (disease duration >2 years). Thus, it may be reasonably speculated that at the initial stage of the disease, WM functional impairments and their underlying structural damages were primarily caused by axon size reductions due to, for instance, demyelination, but as the pathology evolved, reductions in fiber density resulting from axon loss became the main culprit. Of a particular clinical relevance, the transition from fiber dimension to density reductions practically defines a critical point of pathological progression, which may be used as a potential indicator of the nature and severity of WM functional impairments to optimize treatment plans.

The diagnostic value of the identified WM functional signatures is reflected in their high capability in discriminating MSA-C patients and healthy controls. Using the WM bundles that exhibited significant differences between the two groups, we were able to achieve an overall diagnostic efficacy of 94% and 86% for early- and nonearly-stage MSA-C respectively, which offers the potential of using functional features of brainstem WM as a new biomarker for diagnosis of this disease. Furthermore, detailed classification of early- and nonearly-stage disease on the basis of functional signatures of the identified WM bundles found that the sensitivity, specificity and area under curve were all superior for the early-stage patients. The high performance is likely because, during the early-stage, functional alterations of WM bundles with reduced volume were still sensitively detectable by fMRI, whereas during the nonearly-stage, the WM atrophy was primarily contributed by axonal loss, thus creating difficulties for measuring WM function reliably. To date, the diagnosis of MSA particularly during early-stage has been a great challenge to the clinicians, for which many studies have been pursued to enhance the accuracy. Compared to these studies, the superior performance of our classifications based on WM fMRI in identifying early-stage MSA-C allows highly sensitive diagnosis, and, by incorporating fiber dimension and density information, could provide opportunities for timely and effective therapeutic interventions^45^. As a word of caveat, we note that the difference in WM functional signatures between the early- and nonearly-stages, as well as that in structural measures we observed, might be attributable to the age difference between the two groups since the nonearly-stage patients were ~6 years older. However, the age difference alone did not seem to be able to account for the observed functional and structural differences between the two groups because the nonearly-stage group would then be better discriminated from the NCs, just opposite to what we have found.

Lastly but not least importantly, this study found functional abnormalities of certain WM bundles were related to early manifestations of the disease. Specifically, for the early-stage patients, UMSARS-I was demonstrated to be significantly correlated with a combination of the left CST and right ICP, which accounted for ~54% of its total variabilities, and UMSARS-II was significantly correlated with the left CST, which explained ~38% of the variabilities in UMSARS-II. Our findings are subtly different from previous DTI studies^46^, which reported that the SARA score correlated significantly with FA in cerebellar WM, the MCP and posterior limb of the internal capsule. One possibility for this discrepancy is that fMRI and DTI probe different biophysical processes in brain tissue on the basis of different signal contrasts and thus have different sensitivities. In addition, severe damages to, or loss of, WM structures could lead to difficulties for robustly detecting WM function, so that no significant correlations were found between clinical scores and WM abnormalities for the patients in later stages. Nevertheless, the correlations found between functional abnormalities of WM and early clinical manifestations suggest that WM signals may encode behavioral information, which has important implications for clinical evaluations of the disease.

It should be mentioned that this research comes with a few limitations. First, the sample size used in this study was relatively small, so that the conclusion drawn here should be viewed cautiously. To address the sample size issue, we are making efforts to recruit a larger number of subjects for our continued studies. Second, neuroimaging measures were compared only cross-sectionally between individuals with MSA-C and healthy controls, with no longitudinal data being collected and analyzed. In our subsequent research, follow-up studies of individuals with MSA-C will be conducted, along with subject-specific interventional therapies administered on the basis of neuroimaging findings. Third, the diagnosis of MSA-C in the study was based on clinical characterization and careful laboratory tests without pathologic markers and postmortem confirmation. The possibility of misdiagnosis in some patients could not be excluded. Further longitudinal follow-up and studies including large MSA-C cohorts are needed. Lastly, some confounding factors that exist in fMRI studies of GM tend to be more pronounced in WM. These include potential effects of partial volume averaging, limited temporal resolution of fMRI signals, or contaminations from subject’s head motion, scanner signal drifting, and cardiopulmonary fluctuations. Notwithstanding extensive efforts that have been made to minimize the signal contaminations, they could not be eliminated from the fMRI data in their entirety^47^. These confounds may add some uncertainties on the interpretations of the findings from this work.

In summary, this study demonstrates that brain WM in MSA-C patients experiences both functional and structural alterations that can be sensitively detected with MRI. The WM abnormalities are accompanied by impairments to fiber microstructure, which indicates intimate and complex brain structure-function relations in the disease evolution. The diagnostic value of WM functional signals is attested by their capability to differentiate early-stage patients from healthy controls, thus offering the potential of guiding therapeutic interventions to curb the disease progression timely. Our experiments on this neurodegenerative disorder suggest that a technology to non-invasively characterize functional signals in WM holds the promise of substantially extending current approaches to brain network analysis, which could foster novel mechanistic insights into degenerative diseases in WM.

## Methods

### Participants

From July 2019 to March 2021, 30 MSA-C patients and 19 NCs were recruited in this study. Among them, the patients were recruited from the Neurology Department of Xuanwu hospital in Beijing, China (age 57.6 ± 7.3 years; 13 females). Age- and sex-matched NCs were enrolled from the community (age 57.5 ± 8.9 years; 12 females). Diagnoses were performed by experienced neurologists and inclusion criteria for patients were clinical diagnosis of possible or probable MSA according to consensus criteria^1^. Early-stage MSA was defined as duration of disease ≤2 years based on ~25% of the median survival from the European (EMSA-SG)^48^ and North American MSA study groups (NAMSA-SG)^49^. Longer duration (disease duration >2 years) were divided into nonearly-stage MSA-C.

The exclusion criteria were as follows: (1) current or previous history of other nervous system or systemic diseases that might affect central nervous system integrity; (2) similar disorders in 1st- and 2nd-degree relatives; (3) established acquired cause of ataxia; (4) evidence of a significant cognitive deficit (Mini-Mental State Examination score ≤24); (5) contraindications for MRI. These NCs had a normal neurological examination without the history of neurological or psychiatric illnesses or head injury. Motor disabilities in MSA patients were examined using the Motor Examination scores of the Unified MSA Rating Scale, Part I (UMSARS-I) and Part II (UMSARS-II) and SARA. This study was approved by the Medical Research Ethics Committee of Xuanwu Hospital of Capital Medical University. All patients and NCs provided written informed consent before participating in the study.

### MRI data acquisitions

All participants were imaged on a 3.0 T MRI scanner (Magnetom Skyra, Siemens, Germany). Three-dimensional T_1_-weighted images were obtained for each subject using magnetization-prepared rapid gradient-echo sequences with repetition time (TR) = 2530 ms, echo time (TE) = 2.98 ms, flip angle = 7°, voxel size 1.0 × 1.0 × 1.0 mm^3^, field of view (FOV) = 256 × 256 mm^2^. Resting-state fMRI signals were also acquired using gradient-echo type echo-planar imaging (EPI) sequences: TR = 2000 ms, TE = 30 ms, flip angle = 90°, matrix = 64 × 64, voxel size = 3.5 × 3.5 × 3.5 mm^3^, gap = 0 mm, number of slices = 32, and 200 time points. During resting-state scanning, subjects were instructed to relax with their eyes closed without falling asleep. Additionally, diffusion weighted imaging (DWI) data were acquired with the following parameters: TR = 11800 ms, TE = 87 ms, flip angle = 90°, matrix = 128 × 128, voxel size = 1.8 × 1.8 × 2 mm^3^, gap = 0 mm, number of slices = 68. Diffusion sensitized signals were obtained by using thirty non-collinear diffusion weighting directions with *b* = 1000 s/mm^2^ and one image with *b* = 0.

### MRI data processing

#### Resting-state fMRI data analyses

The functional images were preprocessed using the statistical parametric mapping (spm12, https://www.fil.ion.ucl.ac.uk/spm). First, the images were corrected for slice timing and subject’s head motion. Then the anatomical reference images were segmented into GM and WM, which were registered to the mean functional images resulting from the correction procedure. The functional images were then normalized into the Montreal Neurological Institute (MNI) space, along with the coregistered anatomical images as well as the GM and WM segments. Third, the normalized functional images were spatially smoothed with a 4 × 4 × 4 mm^3^ full-width, half-maximum Gaussian kernel. To eliminate potential signal drifting due to scanner imperfection, linear regression of the time courses in the normalized fMRI data was performed voxel-wise. Finally, the time courses were filtered by a band-pass filter (0.01–0.1 Hz).

To analyze functional signals, the WM of each subject was parcellated into 48 regions of interest based on JHU ICBM-DTI-81 WM atlas^50^, which included 21 bundles in each hemisphere and six commissure bundles; GM was parcellated into 82 regions of interest on the basis of Brodmann’s definitions, which included 42 regions in each hemisphere. Furthermore, all WM bundles were restricted within each subject’s WM mask thresholded tightly at 0.95, so as to avoid partial volume effects from nearby GM regions. The functional signals were averaged across each of the GM regions and WM bundles defined above to produce a mean time series, which was used for pairwise temporal correlations between these WM bundles and GM regions. In addition, the PSD of each mean time series was calculated (using Welch’s overlapped segment averaging estimator with 200 s window length and 50% overlap), which yielded the contribution of each frequency component to the total BOLD signals. The frequency range (0.01–0.08 Hz) of each PSD was equally divided into low, medium, and high frequency band and the ratio of each of the three frequency bands to the total frequency range was computed for further analysis.

#### DWI data analyses

The diffusion images were processed with a pipeline toolbox for analyzing brain diffusion images^51^, which reconstructs DTI data, computes diffusion metrics and performs TBSS for statistical inferences. Briefly, DTI data were processed by the following steps: (1) estimate the brain mask and remove the skull from b0 images; (2) crop the raw image and remove non-brain tissue; (3) correct eddy current and motion effects; (4) reconstruct diffusion tensor; (5) generate MD and FA maps; (6) perform TBSS for the voxel-wise analysis of FA and MD. A mean FA map was obtained by averaging the FA maps from all MSA subjects in the MNI space. The skeleton of the mean FA map was computed and thresholded at 0.2 to include only voxels indicative of WM which representing the study-specific centers of all fiber tracts. Then, the individual FA maps were projected onto the FA skeleton to obtain the FA skeletons of each participant and the deformation matrixes. The projection information was also applied to the MD maps and the resulting skeletonized FA and MD maps were used for further statistical analyses. In addition, within-voxel fiber density and macroscopic fiber-bundle cross-sectional area were calculated using fixel- based analysis^42^, with detailed procedures described in Supplementary Material F.

### Classifications of MSA-C patients

Functional correlations of WM bundles were used to classify early-stage, nonearly-stage MSA-C patients and NCs. Specifically, a conventional classifier, SVM^52^, was employed for discriminant analysis, and the features for the SVM classifier were the average WM functional correlation in regions showed significantly difference between the patients and NCs. In this work, discriminant analysis was carried out between each pair of the three subject groups above. For each classification, a cross-validation was applied to divide the sample dataset into two complementary subsets, one as a training set, and the other for verifying the validity of the analysis as a testing set. To reduce the variability of classification, leave one out cross-validation was performed^52^. The sensitivity, specificity and AUC were calculated to quantify the classification performance, with larger values indicating better performance.

### Statistical analysis

Demographic characteristics were compared between patients and NCs using two-tailed *t* tests. Two-sample *t* tests were also performed to compare correlation coefficients of fMRI signals in the parcellated WM bundles and GM regions between the patients and NCs. Multiple comparisons were corrected by using the two-sample *t* tests with a false-positive correction *P* < (1/48) for WM bundles and *P* < (1/82) for GM regions, as previously implemented^53^. Note that this correction approach does not require independency among the *t* tests; it is not as conservative as Bonferroni correction, and allows only one false-positive regardless of the number of *t* tests. For DTI statistics, a voxel (TBSS Randomize) analysis based on the FA and MD images was conducted between the patients and NCs. Significant differences were estimated with 500 random permutations using threshold-free cluster enhancements (TFCE) and family-wise error (FWE) correction for multiple comparisons. The significance threshold was *P* < 0.05 (TFCE and FWE corrected). Whole brain fiber density and fiber-bundle cross-section area were also compared between the patients and NCs, with significant differences estimated with 5000 permutations using non-parametric permutation testing and FWE corrected *p* value^54^. To explore whether WM functional signals could predict subject’s behavior, multiple stepwise linear regression analyses were employed to examine the relation of WM functional signals to clinical variables (UMSARS-I, UMSARS-II, and SARA) in the patient group.

## Supplementary information

Supplementary Material A-F

## Data Availability

All data generated and analyzed in the current study were collected from distinct subjects and are available from the corresponding author upon reasonable request.

## Code availability

The study was developed based on Matlab (MathWorks, Inc) R2016b and R(version 3.6.2) using a combination of freely available tools listed below and custom code. SPM12, https://www.fil.ion.ucl.ac.uk/spm. For fMRI preprocessing, we used Data Processing Assistant for Resting-State fMRI (DPARSF, http://www.rfmri.org/DPARSF). For DTI preprocessing, we use Pipeline for Analyzing brain Diffusion images (PANDA,https://www.nitrc.org/projects/panda/) and MRtrix3 (https://github.com/MRtrix3).
