# Supplementary material for: Combined functional and structural imaging of brain white matter reveals stage-dependent impairment in multiple system atrophy of cerebellar type
Source: NPJ Parkinsons Dis. 2022 Aug 17;8:105. doi: 10.1038/s41531-022-00371-2 (PMC9385720; doi:10.1038/s41531-022-00371-2)
Supplement: Supplementary file 1 — Supplementary Material A-F [file 41531_2022_371_MOESM1_ESM.pdf]

Combined functional and structural imaging of brain white matter reveals stage-dependent impairment in multiple system atrophy of cerebellar type

Hua Lin [1† *], Li Lin [2†], Lyuan Xu[3,4], Siran Li[1], Penghui Song[1], Muwei Li[3,4]

[†]These authors contributed equally to this work.

Author affiliations:

1 Department of Neurology, Xuanwu Hospital, Capital Medical University, Beijing 100053, China

2 Department of Radiology, the Third Affiliated Hospital of Sun Yat-sen University, 600 Tianhe Road, Guangzhou, Guangdong Province 510630, China

3 Vanderbilt University Institute of Imaging Science, Vanderbilt University, Nashville, TN,USA

4 Department of Radiology and Radiological Sciences, Vanderbilt University Medical Center, Nashville, TN, USA

[*] Correspondence to: Hua Lin

Department of Neurology, 45 Changchun Street, Xicheng District, Beijing 100053, China.

E-mail: linhua@ccmu.edu.cn.

Supplementary Material A

List of abbreviations for white matter bundles

Abbreviations	Full name of WM bundles
CST	Corticospinal tract
ML	Medial lemniscus
ICP	Inferior cerebellar peduncle
SCP	Superior cerebellar peduncle
CP	Cerebral peduncle
ALIC	Anterior limb of internal capsule
PLIC	Posterior limb of internal capsule
RLIC	Retrolenticular part of internal capsule
ACR	Anterior corona radiata
SCR	Superior corona radiata
PCR	Posterior corona radiata
PTR	Posterior thalamic radiation (include optic radiation)
SS	Sagittal stratum
EC	External capsule
CGC	Cingulum (cingulate gyrus)
CGH	Cingulum (hippocampus)
Fx/ST	Fornix (cres) / Stria terminalis
SLF	Superior longitudinal fasciculus
SFO	Superior fronto-occipital fasciculus
UNC	Uncinate fasciculus
TAP	Tapetum
BCC	Body of corpus callosum
SCC	Splenium of corpus callosum
Fx	Fornix (column and body of fornix)

Supplementary Material B

Power spectra analysis

In Supplementary Figure 1, (**a**) and (**b**) are for the control and entire patient group, (**c**) and (**d**) are for early- and nonearly-stage sub-group respectively. Grossly, compared to the control subjects, the WM bundles of the patients tended to have lower PSD in the high frequency band (0.056 – 0.080 Hz), and higher PSD in the medium frequency band (0.033 – 0.056 Hz). Analyses based on stratification of the patients into two sub-groups of different disease durations show that early-stage patients had a smaller PSD ratio in the high frequency band in right cingulum and greater PSD ratio in the medium frequency band in the MCP, both of which reached a statistically significant level of $P < 0.05$ (two sample t-tests). Meanwhile, nonearly-stage patients also showed a smaller PSD ratio in right cingulum but greater PSD ratio in left ICP in the high frequency band. Detailed statistical tests are reported in Supplementary Table 1. These alterations in PSD may underlie the abnormalities in correlation profiles of the WM bundles observed in the MSA-C patients.

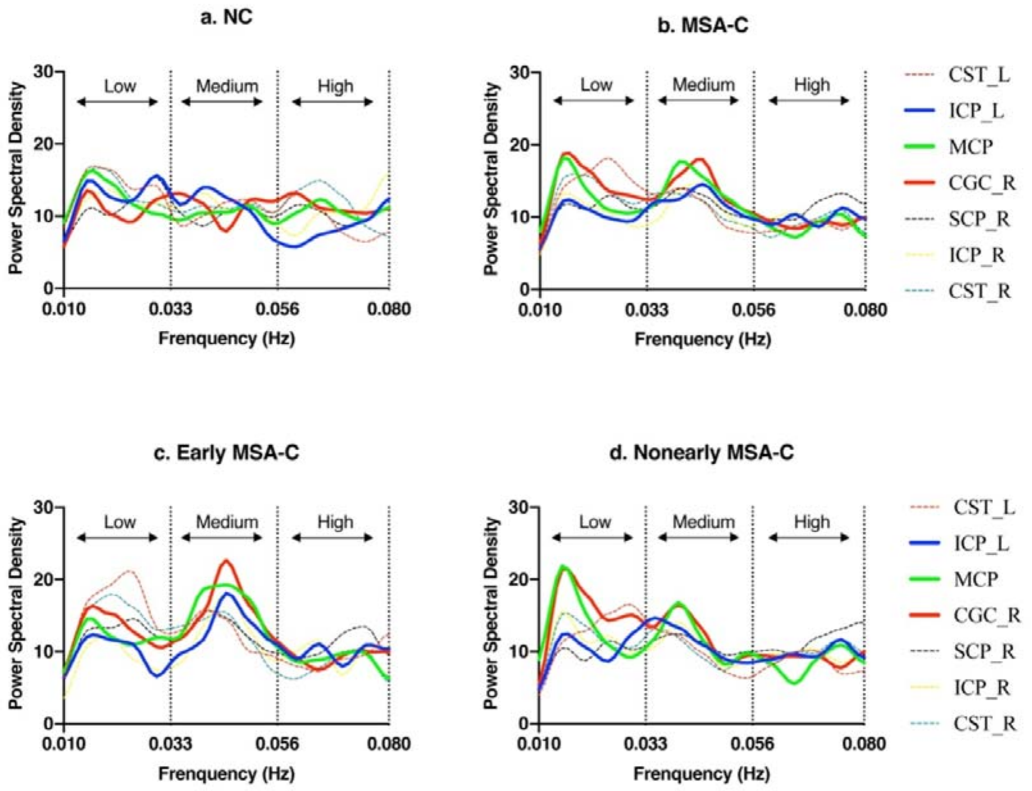

Supplementary Figure 1. Power spectra of white matter fMRI signals between normal controls and MSA-C patients. **(a)-(d)** Power spectral densities of fMRI signals of the WM bundles in (A) with significant between-group differences, respectively for the NCs, entire MSA-C group, early- and nonearly-stage sub-groups. It can be seen that, compared to the NCs **(a)**, the MSA-C patients exhibited qualitatively different PSD profiles **(b)**. Further stratification of the patients on the basis of disease duration

shows that early-stage patients **(c)** tended to have high PSDs in the medium frequency band (0.033 – 0.056 Hz), andhigh PSDs tended to shift toward the low frequency band (0.010 – 0.033 Hz) in nonearly-stage patients **(d)**. Note that WM bundles with significant differences between the normal controls and patient group or sub-groups are denoted with thick solid curves throughout (**a**) to (**d**).

Supplement Material C

Supplementary Table 1 Quantitative comparisons of PSD between normal controls and MSA-C patients

		Subject group and *P* value						
	**WM bundle**	**Normal controls**	**MSA-C patients**	***P* value**	**Early patients**	***P* value**	**Nonearly patients**	***P* value**
High frequency band# (0.056–0.080 Hz)	CST_L	0.431	0.404	0.755	0.347	0.341	0.461	0.781
	ICP_L	0.361	0.469	**0.032***	0.458	0.095	0.480	**0.033***
	MCP	0.591	0.370	**0.029***	0.344	0.058	0.397	0.159
	CGC_R	0.553	0.334	**0.003***	0.324	**0.016***	0.343	**0.030***
	SCP_R	0.553	0.570	0.864	0.515	0.774	0.626	0.555
	ICP_R	0.529	0.486	0.632	0.492	0.736	0.480	0.648
	CST_R	0.523	0.467	0.614	0.352	0.108	0.581	0.684
Medium frequency band# (0.033–0.056 Hz)	CST_L	0.468	0.519	0.470	0.521	0.404	0.517	0.584
	ICP_L	0.558	0.689	0.256	0.779	0.126	0.598	0.705
	MCP	0.479	0.703	**0.039***	0.822	**0.006***	0.583	0.364
	CGC_R	0.544	0.694	0.224	0.847	0.057	0.541	0.974
	SCP_R	0.490	0.597	0.324	0.626	0.238	0.569	0.514
	ICP_R	0.554	0.632	0.430	0.727	0.131	0.537	0.876
	CST_R	0.492	0.587	0.309	0.629	0.194	0.544	0.597
Low frequency band# (0.010–0.033 Hz)	CST_L	0.788	0.808	0.884	0.846	0.720	0.770	0.915
	ICP_L	0.769	0.535	0.065	0.525	0.148	0.544	0.139
	MCP	0.731	0.630	0.459	0.527	0.230	0.733	0.990
	CGC_R	0.554	0.722	0.162	0.623	0.608	0.820	0.065
	SCP_R	0.619	0.536	0.413	0.609	0.941	0.463	0.102
	ICP_R	0.622	0.552	0.575	0.436	0.242	0.667	0.785
	CST_R	0.633	0.737	0.516	0.856	0.284	0.619	0.904

# denotes the ratio of a frequency band to the total frequency range. ***Boldface** denotes significant difference ($P < 0.05$).

Supplementary Material D

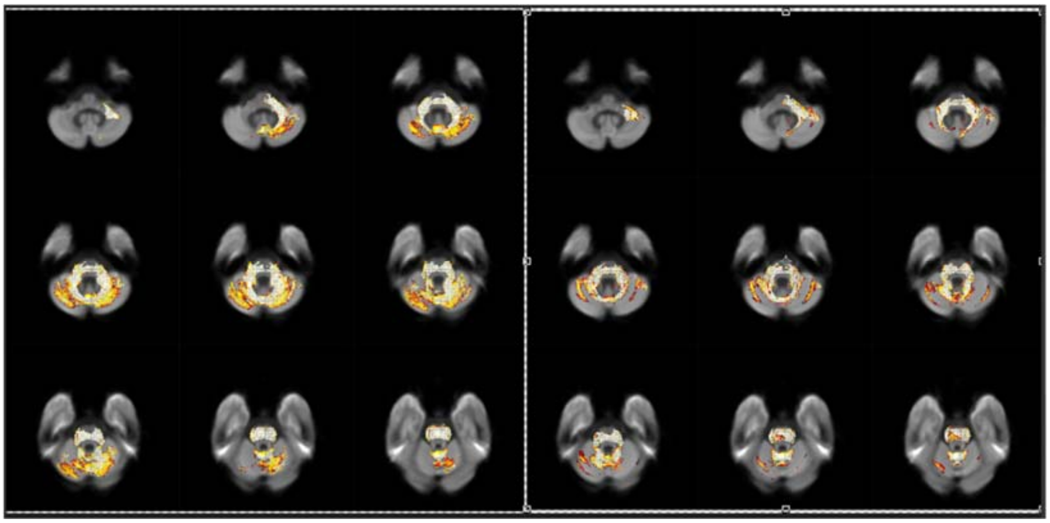

Supplementary Figure 2. Superposition of fiber-bundle cross-sectional area and fiber density of early-stage (left panel) and nonearly-stage (right panel) MSA-C patients

Supplementary Material E

Evaluation of clinical significance of white matter functional alterations

Our stepwise linear regression analyses showed that, for the early-stage patients, there was a significant strong relationship between UMSARS-I and a combination of left CST and right ICP (r = 0.736, P = 0.009), and a significant moderate relationship between UMSARS-II and left CST (r = 0.613, P = 0.015). No significant relationships were found for the nonearly-stage patients. These findings demonstrated that fMRI signals in the impaired WM bundles were able to largely predict the severity of the disease during its initial progression phase, but when the severity is beyond a certain level, the alterations in WM functional signals had no relationships with the clinical performances.

Supplementary Material F

The fixel-based analysis used in this work involves a series of image preprocessing and processing steps, which are outlined below:

(1) denoising and unringing;

(2) motion and distortion correction;

(3) bias field correction;

(4)global intensity normalization across subjects;

(5) computation of an average white matter response function;

(6) upsampling of diffusion images;

(7) computation of upsampled brain mask images;

(8) estimation of fiber orientation distribution (FOD);

(9)generation of a study-specific unbiased FOD template;

(10) registration of all subject FOD images to the FOD template;

(11) computation of a common mask by intersecting individual subject masks in the template space;

(12) computation of a voxel mask for white matter template analysis;

(13) warping of FOD images to the template space;

(14)segmentation of FOD images to estimate within-voxel fiber density andmacro-scopic fiber-bundle cross-sectional area.
